# Generative learning activities for online multimedia learning: when summarizing is effective but drawing is not

**DOI:** 10.3389/fpsyg.2024.1452385

**Published:** 2024-09-02

**Authors:** Alyssa P. Lawson, Richard E. Mayer

**Affiliations:** ^1^Institute for Research and Training, Landmark College, Putney, VT, United States; ^2^Department of Psychological and Brain Sciences, University of California, Santa Barbara, Santa Barbara, CA, United States

**Keywords:** drawing, generative learning activities, online learning, summarizing, combining activities

## Abstract

**Introduction:**

The goal of this study is to determine whether two commonly used generative learning activities for text-based lessons—writing a summary or creating a drawing—help students learn from a multimedia lesson involving animations with short text captions without prior training in the generative activities.

**Methods:**

Students viewed a series of four annotated animations on greenhouse gases. During pauses between the animations, students were asked to generate a written summary, to create a drawing, or to do both, whereas a control group viewed the lesson without any generative learning activities. Students were tested immediately (Experiment 1) or after a one-week delay (Experiment 2).

**Results:**

In both experiments, students who produced written summaries scored significantly higher on the posttest than those who engaged in no generative learning activities (*d* = 0.48 in Experiment 1, *d* = 0.54 in Experiment 2), but there was no significant difference on the posttest for students who generated drawings compared to those who engaged in no generative learning activities. In addition, those who engaged in drawing and summarizing did not have significantly different posttest performance than those engaged in summarizing alone.

**Discussion:**

We conclude that writing summaries during a highly visual animated lesson is effective for learning, possibly because it encourages students to engage in generative processing during learning more than drawing and we discuss potential reasons for this in the discussion. This work helps extend generative learning theory by pinpointing potential boundary conditions for learning by drawing and learning by summarizing.

## Introduction

### Objective and rationale

**Figure 1:**
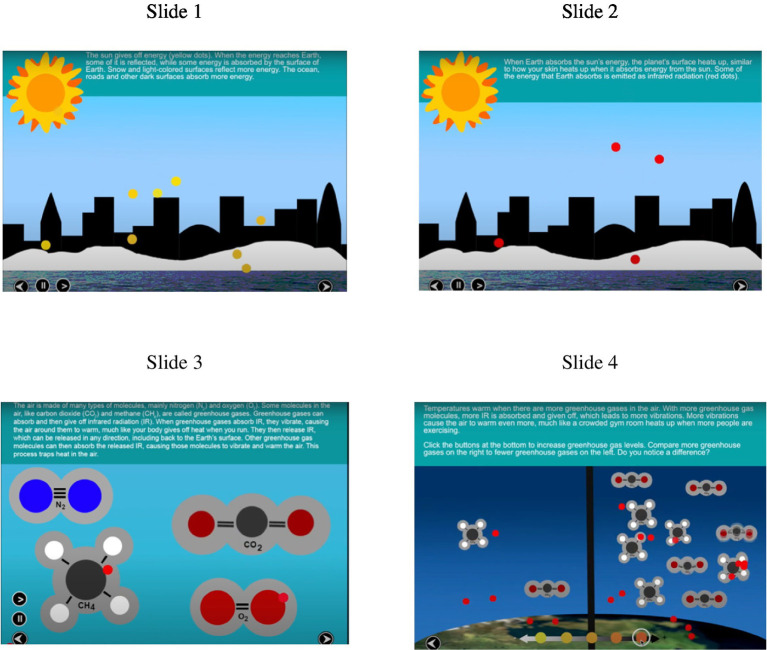
Still screenshots from the lesson.

Multimedia lessons involving animation and video are prevalent in education, especially in online formats in which instructors are not directly monitoring students to ensure learning (Mayer et al., 2020). For example, Figure 1 shows screenshots from a lesson explaining how greenhouse gases impact the environment through a series of simple animations with short, printed explanations as captions. As remote learning, particularly asynchronous learning, becomes more widely used, it is important to understand how to make sure that online multimedia lessons are effective in promoting deep learning. Deep learning occurs when learners engage in active cognitive processing during instruction by attending to the relevant information, mentally organizing it into a coherent structure, and relating it to other knowledge structures and to relevant prior knowledge (Mayer, 2021). Deep learning is indicated by transfer test performance in which learners can apply what they have learned to solve new problems or answer new questions. One way to encourage students to process information more deeply from online multimedia lessons is to incorporate generative learning activities during learning, that is, activities students perform during a lesson that are intended to foster their learning (Brown et al., 2014; Dunlosky et al., 2013b; Fiorella and Mayer, 2015, 2016; Mayer, 2021; Miyatsu et al., 2018).

Two common generative learning activities, generally used with lessons containing printed text, are summarizing—in which the learner is asked to produce a brief written or oral summary of a portion of a lesson—and drawing—in which the learner is asked to produce an illustration that depicts a portion of a lesson (Fiorella and Mayer, 2015, 2016). Van Meter and Stepanik (2020) refer to these kinds of in-lesson prompts to engage in generative activities as prompt-driven interventions. In reviews, both techniques have been shown to be effective across multiple studies: summarizing was superior to control conditions in 26 of 30 experiments with a median effect size of *d* = 0.50 whereas drawing was superior to control conditions in 26 of 28 studies with a median effect size of *d* = 0.40 (Fiorella and Mayer, 2015). However, almost all of these studies are based on lessons that rely exclusively or predominately on printed text, so less is known about how summarizing and drawing work with multimedia lessons that are heavily visual, such as lessons that involve animations with short text captions. The present study aims to help to fill this gap in the literature.

As a field matures, the focus shifts from whether a particular learning activity is effective (i.e., what works) to determining the boundary conditions under which it works (i.e., when it works). This study delves deeper into the role of generative learning activities by examining which activity—summarizing or drawing—is more effective for improving learning from multimedia lessons involving simple animations with short text captions. We focus on applying summarizing and drawing without providing students with prior training in the strategy, as this is a likely scenario learners will find themselves in some online learning situations. Our justification for not including prior training is that we were interested in the effectiveness of generative learning activities that require minimal intervention. Learning from annotated animations (which can be called heavily visual material) is a different media context than learning from printed text (which can be called heavily verbal material). Thus, it is worthwhile to determine the conditions under which summarizing and/or drawing is effective.

An important aspect of generative learning activities is that they encourage learners to translate instructional material from one form of representation to another, which promotes active cognitive processing (Ainsworth, 2022; Ainsworth et al., 2020; Fiorella and Mayer, 2015, 2022; Mayer, 2021). In the present study, summarizing involves translating from a lesson that is heavily visual to a verbal form of representation whereas drawing involves translating from a lesson that is heavily visual to another visual form of representation. Although both generative learning activities can encourage students to create a translation, students may not feel the need to work as hard at translating when moving from one representation to the same representation (i.e., visual to visual) than when moving from one representation to a different representation (i.e., visual to verbal). This could be particularly problematic in an environment where students are not monitored by an instructor, like in an online, self-paced lesson. As such, we suspect that summarizing will be effective in improving posttest performance whereas drawing may not be effective in an online, self-paced environment. As translation from one form of representation to another is thought to be a key component of developing a deeper understanding of lesson content, some generative learning activities may be better suited to encourage translation than others (Fiorella and Mayer, 2015). Thus, our goal is not only to contribute to the replication research base—which is a recognized, fundamental contribution of scientific research in education (Shavelson and Towne, 2002)—but also to contribute new findings concerning the boundary conditions of when summarizing and drawing are effective learning activities.

Although there is a substantial research base on generative learning activities for learning from printed text (e.g., Dunlosky et al., 2013b; Fiorella and Mayer, 2015, 2016, 2022; Lee et al., 2009; Miyatsu et al., 2018), there is less known about how generative learning activities work in online, self-paced multimedia learning venues involving dynamic graphics such as animations with minimal text. In particular, given the wide array of generative learning activities, there is a need for research aimed at determining which generative learning activities can be effective without prior training in strategy use and without intervention from an instructor. In short, we sought minimal interventions implemented without prior training to encourage generative processing in online multimedia learning situations that are heavily visual, especially with the increase of self-guided learning material being available to students online. The present study seeks to address this need by examining how learning from an annotated animation on greenhouse gases is affected by asking students to summarize, draw, or do both during pauses in the online lesson.

Furthermore, an aim of this paper is to investigate how the impact of drawing, summarizing, and drawing combined with summarizing impacts posttest performance on both an immediate and delayed test. We are particularly interested in this question as many studies focus on impacts of generative learning activities with immediate testing, but typically educational environments involve delayed testing (Fiorella and Mayer, 2015, 2016). Some previous work suggests that generative learning activities promote meaningful learning, and this type of learning shows up better for delayed tests than immediate tests (e.g., Katona, 1942; Mayer and Wittrock, 2006; Wertheimer, 1959). In some cases, impacts of similar types of activities do not have positive impacts on immediate tests but do on delayed tests (e.g., Brown et al., 2014; Dunlosky et al., 2013b; Fiorella and Mayer, 2015, 2016; Roediger III and Karpicke, 2006). For these reasons, we examined the effects of generative learning activities on both immediate tests and delayed tests in the present study.

There are two major goals of this research. The first aim is to understand whether the different tactics used by summarizing and drawing, without previous training on the learning strategy and without intervention from an instructor, encourage generative learning from an online, self-paced multimedia lesson and lead to a better understanding of the material presented. The second aim of this research is to understand whether combining two generative learning activities can encourage more generative learning and produce better learning outcomes than asking learners to engage in only one activity. These aims represent emerging new trends in multimedia research and add to the fledgling research base on extending generative learning activities developed mainly for text-based lessons to online multimedia lessons involving animation with short text captions without guidance from an instructor. Finally, as a secondary aim, this paper also investigated if the impact of learning from drawing and/or summarizing changed depending on when the posttest occurred.

### Literature review

#### Learning by summarizing

Summarizing occurs when a learner restates the main ideas of a lesson in their own words (Fiorella and Mayer, 2015, 2016). This is considered a generative learning activity because students are prompted to engage with selecting, organizing, and integrating the material to create a summary. First, students must select the ideas they want to include in their summary. Next, the student must organize those ideas into a cohesive representation of the material. Lastly, they must integrate these ideas with what they already know to put the summary in their own words. Although the goal of summarizing is to prime students to engage in selecting, organizing, and integrating as they learn, summarizing is a verbal tactic that relies mainly on processing in the learner’s verbal channel of the text (Leopold and Leutner, 2012). Summarizing can be done in two ways: while students have access to the learning material, referred to as *during learning*, or after all the learning content has been presented, referred to as *after learning* (Fiorella and Mayer, 2015, 2016). For the purposes of this study, we focus on summarizing during learning rather than on summarizing after learning as summarizing after learning can be seen as more of a testing activity, which has separate benefits outside of summarizing (Fiorella and Mayer, 2015, 2016).

In much of the early research on summarizing, students were asked to summarize during learning when reading printed text in order to understand the material better by engaging in selecting, organizing, and integrating. For example, students who were asked to generate one-sentence summaries of each paragraph of short stories during learning performed better on comprehension tests than students who were not asked to summarize (Doctorow et al., 1978). In another study, students were asked to create summaries of a text-based history lesson, take notes, or simply read the material (Annis, 1985). Those who created summaries of the material performed better on delayed posttests assessing application and analysis levels compared to students who were asked to go through the text as they normally would or take notes. Other research has found similar benefits of asking learners to generate verbal summaries during learning of text-based lessons (e.g., Hooper et al., 1994; King et al., 1984; Wittrock and Alesandrini, 1990).

Generally, there is a positive impact of summarizing on learning from text, with a meta-analysis investigating summarizing demonstrating small but positive effects (Donoghue and Hattie, 2021). However, not all studies on summarizing yield positive results (Donoghue and Hattie, 2021; Dunlosky et al., 2013a; Leopold and Leutner, 2012; McNamara et al., 2024). Although not specifically investigated, this may be due to the type of knowledge that learners are asked to remember (e.g., application knowledge seems to benefit from summarizing while evaluation knowledge does not), the type of test that is given (e.g., open-ended exams seem to show benefits of summarizing while multiple-choice exams do not), or knowledge on how to summarize effectively (e.g., those who know how to summarize seem to benefit more from this technique than those who do not; Dunlosky et al., 2013a; Fiorella and Mayer, 2015). In light of the variability in how summarizing has been implemented across studies, it is difficult to draw conclusions without further investigation into what specifically was done in each study, as mentioned by both Dunlosky et al. (2013a) as well as Donoghue and Hattie (2021).

More recently, researchers have examined how to apply summarizing to multimedia learning venues (Lawson and Mayer, 2021; Parong and Mayer, 2018; Wang et al., 2023). For example, Lawson and Mayer (2021) found that students performed better on a posttest when students wrote summaries explaining the lesson during pauses in an online multimedia lesson on greenhouse gases compared to a group that viewed a lesson with no generative learning activities during pauses. Parong and Mayer (2018) reported that students who were asked to summarize during breaks during an immersive virtual reality (IVR) lesson on the human bloodstream performed significantly better on a posttest than students who did not summarize during breaks in an IVR lesson. Wang et al. (2023) found that asking students to write or imagine writing summaries during pauses between slides of a lesson enhanced both retention and transfer posttest scores while providing a summary to students during the same time period only helped with retention.

This work demonstrates how there is variability in the impact of using summarizing to improve learning, in both text-based and multimedia learning environments. Although many studies show the benefits of using generative learning activities, not all studies do. This suggests that there are likely boundary conditions that need to be investigated further to understand when and how summarizing can be effectively used.

#### Learning by drawing

Learning by drawing occurs when learners are prompted to draw a visual representation of the material they are learning about (Fiorella and Mayer, 2015, 2016). Learning by drawing is considered a generative learning activity because it prompts students to engage in the cognitive processes of selecting, organizing, and integrating. It prompts students to select the information they want to draw from the studied material. Then, they must organize that information into a coherent drawing. Lastly, they must integrate their new knowledge with their prior knowledge in order to translate their understanding of the material into a drawing that conveys a meaningful message. In contrast to summarizing, which requires creating a verbal representation, drawing prompts learners to develop a visuo-spatial representation of the presented material (Leopold and Leutner, 2012; Van Meter and Garner, 2005; Wu and Rau, 2018). In short, drawing involves a visual tactic to prime relevant cognitive processes during learning, in which learners translate the presented information into the visuo-spatial drawing.

Some research has demonstrated that simply including prompts to draw from expository text can promote transfer learning (e.g., Leopold and Leutner, 2012; Lin et al., 2017; Schmeck et al., 2014; Schmidgall et al., 2019; Schwamborn et al., 2010; Van Meter, 2001). For example, Schwamborn et al. (2010) asked students to generate drawings after reading a text about the chemical process of using soap in laundry or simply read the text. Students who drew representations of the text performed significantly better on transfer and retention tests. Additionally, the quality of the drawings the students created was correlated with their test performance, with students who produced higher quality drawings doing better on the posttest. Similarly, students who were asked to draw during learning about the biology of influenza outperformed students who only read the text on a comprehension test (Schmeck et al., 2014). Many studies have found similar benefits of asking students to generate drawings to accompany text-based lessons (e.g., Leutner and Schmeck, 2022).

There has been relatively less research on how the generative learning activity of drawing can be used in multimedia lessons (Leutner and Schmeck, 2022; Fiorella and Mayer, 2015; Mason et al., 2013; Zhang and Linn, 2011). One study demonstrated the benefits of having students create drawings throughout a multimedia lesson, specifically on a delayed test, possibly because students were able to revise their drawings (Wu and Rau, 2018). Another study had student generate drawings from an animation, copy the drawing, or not draw (Mason et al., 2013). Those who were asked to generate their own drawings had significantly better comprehension of the material on both immediate and delayed tests. Those who copied the drawings had similar comprehension to the no drawing condition.

However, a meta-analysis on the impact of drawing has shown that drawing from animations does not seem to be as effective in promoting learning as drawing from text (Cromley et al., 2019). This may be because drawing from a multimedia lesson does not always encourage learners to transform information from one mode of representation to another, whereas in drawing from text, learners have to transform information from a verbal mode to a pictorial mode (Ploetzner and Fillisch, 2017; Van Meter and Firetto, 2013; Van Meter and Garner, 2005). As with the results on summarizing, the mixed results on drawing suggest that more research is warranted on the conditions under which adding drawing prompts in multimedia lessons can enhance learning. A boundary condition for when drawing is not effective may be an environment in which learners are not motivated to engage in translation, like with a highly visual lesson that is online and self-paced.

#### Literature on combining generative learning activities

In addition to the research on generative learning activities investigated in isolation from one another, there have been some studies that have looked at the benefit of combining these activities. For example, Leopold and Leutner (2012) asked students to summarize main ideas, draw, do both, or do neither as they read a science text. Although drawing improved comprehension posttest performance, summarizing did not, and combining summarizing with drawing also did not benefit learning. In another study, Ponce et al. (2018) asked students to read a text only, highlight as they read, take notes, use a graphic organizer, highlight then take notes, or highlight then use an interactive graphic organizer. The results of this study found that highlighting and then using a graphic organizer was better for learning than highlighting alone, but not better than using a graphic organizer alone. Additionally, highlighting and then notetaking was better for learning than notetaking alone, but not better than using highlighting alone.

These studies show that there may be some additive effects to combining generative learning activities in specific situations, when one activity builds on another; however, the effect of combining learning activities is not generally a straightforward matter of simply adding individual effects. An important practical and theoretical question concerns the degree to which asking learners to engage in two generative learning activities creates additive effects beyond engaging in just one activity. This is of particular interest for online, self-paced lessons, as the different prompts ask students to translate their knowledge in two different ways and thus should encourage deeper learning. To address this issue, in the present study we examine the consequences of combining two generative learning activities that use different tactics–through focusing on verbal/textual translation and focusing on visual–spatial translation–to promote generative processing in different ways from a multimedia lesson.

### Theoretical framework

This research is grounded in generative learning theory (Fiorella and Mayer, 2015; Mayer, 2021; Wittrock, 1974, 1989). According to generative learning theory, generative learning activities, such as summarizing and drawing, are intended to prime learners to engage in appropriate cognitive processing during learning, including *selecting* (i.e., attending to relevant aspects of the incoming information for further processing in working memory), *organizing* (i.e., mentally organizing the selected material into a coherent structure), and *integrating* (i.e., mentally connecting visual and verbal representations with each other and with relevant knowledge activated from long-term memory). Thus, to the extent that these methods are effective in priming appropriate cognitive processing during learning, generative learning theory proposes that students who are asked to engage in generative learning activities during learning (e.g., summarizing, drawing, or both) will learn better from the present lesson than those who are not asked to engage in generative learning activities. Van Meter and Stepanik (2020) also show how this analysis applies to learning with multiple external representations, including visual and verbal representations.

At a gross level that focuses on the quantity of learning activity, generative learning theory predicts that learners who engage in generative learning activities should perform better on a transfer test than those who do not because these activities should encourage learners to engage in generative processing. In the present study, this means that participants who are prompted to summarize, a generative learning activity, should perform better on a transfer test than those in a control condition, and participants who are prompted to draw, another generative learning activity, should also perform better on a transfer test than those in a control condition. Similarly, participants who are prompted to summarize and draw should perform better on a transfer test than those in a control condition.

However, thinking more deeply about what is occurring cognitively during the generative learning activity with a heavily visual multimedia lessons, there may be differences in the effectiveness of summarizing and drawing. First, when summarizing, students must translate from a heavily visual form of external representation—animation with short captions—to a verbal form of representation—a written summary in order to successfully complete the prompted activity. The act of summarizing is more likely to prompt learners to represent the core material both visually (based on the external presentation) and verbally (based on the generative activity of creating a verbal summary) and to build integrative connections between them. Learning with multiple representations is examined in Ainsworth’s (2022) DeFT model, which focuses on the function of the multiple representations: complementary (in which each contributes unique information), constraining (in which a simple representation supports understanding a complex one), and constructing (in which learners achieve deeper understanding when they integrate two different ways of representing the same thing). The constructing function is similar to the integrating process in the CTML (Mayer, 2021), which posits that people learn more deeply when they make connections between visual and verbal representations.

Additionally, with respect to drawing, students must translate from a heavily visual form of external representation—animations with short captions—to another visual form of representation—a drawing. Although this does not mean that translation will not occur, it could mean that students are less motivated to create a well-develop translation that induces deeper understanding, especially when the drawing process is not monitored by an instructor. In the present study, students may form visual representations but may be less encouraged to integrate them with corresponding verbal representations without prompting from an instructor. This analysis suggests that the summarizing and drawing with annotated animations may have different effects on the processes of selecting essential verbal and visual information, mentally organizing them into coherent verbal and visual representations, and integrating corresponding verbal and visual representations with each other. This is also supported by some literature demonstrating the diminished benefits of using drawing during learning with multimedia lessons (e.g., Ploetzner and Fillisch, 2017; Van Meter and Firetto, 2013; Van Meter and Garner, 2005).

As such, with a more refined level of generative learning theory that focuses on the quality of learning activity as described above, it is predicted that, when learning with an online, self-paced multimedia lesson, summarizing will be more effective than a control group in promoting the cognitive processes of selecting, organizing, and integrating, and thereby result in superior transfer test performance (hypothesis 1a). In contrast, with the more refined level of generative learning activity, it is predicted that when learning with an online, self-paced multimedia lesson, drawing will not be more effective than a control group in promoting transfer test performance because students may not be as encouraged to integrate corresponding visual and verbal representations (hypothesis 1b).

This research is also interested in an exploratory question: do students learn better if they engage in two generative learning activities (summarizing and drawing) rather than one (summarizing or drawing). From the first perspective we mentioned, generative learning theory generally would predict that more activity leads to more appropriate cognitive processing. Thus, learners who engage in two activities should perform better on a transfer test than those who only engage in one activity. More specifically, those who engage in both summarizing and drawing should perform better on a transfer test than those who only engage in summarizing and those who only engage in drawing. However, from the refined interpretation perspective of generative learning theory this paper takes, drawing may not be an effective strategy for this medium, then engaging in two both drawing and summarizing should not result in better transfer test performance than summarizing alone but should result in better transfer test performance than drawing alone if only summarizing is effective (hypothesis 2).

## Experiment 1

Experiment 1 investigated how engaging in summarizing, drawing, or both activities during pauses in a multimedia science lesson affects learners’ performance on an immediate test as compared to a control group that does not engage a generative learning activity.

### Method

#### Participants and design

The participants were 203 undergraduate students recruited from a university in southern California through the university’s psychology subject pool. The mean age of these participants was 19.10 years (*SD* = 1.42). Of the 203 participants, 144 of them identified as women and 59 identified as men. The experiment used a one-way between-subjects design with four levels, including a summary only condition with 55 participants, a draw only condition with 48 participants, a draw and summary condition with 46 participants, and a control condition with 54 participants. Based on previous literature investigating the impact of summarizing and drawing on learning (*d* = 0.50, *d* = 0.40, respectively, Fiorella and Mayer, 2015), an effect size of *d* = 0.45 (*f* = 0.23) was used. According to a power analysis based on G*Power (Faul et al., 2007) with power set at 0.80 and alpha set at 0.05, our sample size was slightly below the required number of participants (which was 212). According to a *post hoc* analysis, the power achieved in this test was 0.78. Results should be interpreted with this power issue under consideration.

#### Materials

All of the materials were provided online through Qualtrics and included instructions, a prequestionnaire, a four-slide multimedia lesson with one of four activities completed after each slide, a distractor task, a posttest, and a postquestionnaire.

##### Instructions

The instructions described how to complete each part of the study. As this study was self-paced and done without any guidance from the experimenters, as a way to mimic asynchronous learning environments, the instructions provided a concrete overview of what the participant would be doing. More specific instructions were also written at the top of each page, reminding participants what they should be doing on each page of the study. Additionally, there was a section that asked participants to practice uploading an image to the Qualtrics page. They were instructed to take a picture of a wall and practice submitting to Qualtrics, as they may be asked to submit images of their paper-based work during this study.

##### Prequestionnaire

The prequestionnaire asked participants to provide information about themselves, including their age and gender. Participants were also asked to rate the knowledge of how greenhouse gases work on a 5-point scale from “Very Low” to “Very High” as a way to assess for subjective prior knowledge. This scale was used to assess knowledge rather than a pretest to minimize the impact of the testing effect, which states the act of taking a test is an instructional event in itself that can cause learning to occur and can influence how the learner processes the lesson (Brown et al., 2014; Roediger III and Karpicke, 2006). This type of assessment for prior knowledge used in this study has been used in similar work previously (e.g., Lawson and Mayer, 2021).

##### Multimedia lesson

All participants saw the same lesson explaining how greenhouse gases warm the atmosphere. The lesson was split into four slides, each containing an animation and short text caption explaining what was occurring in the animation, as exemplified in Figure 1. The first slide explained how energy comes from the sun and is either absorbed by or reflected off the Earth. The second slide explained how absorbed energy warms the Earth which causes a release of infrared radiation (IR). The third slide explained how IR interacts with greenhouse gases, trapping IR in the atmosphere. The fourth slide explained that when there are more greenhouse gases in the atmosphere, the atmosphere becomes warmer. This lesson came from KQED public television’s website, found here: https://ww2.kqed.org/quest/2014/12/12/how-do-greenhouse-gases-work/. Participants were allowed to stay on each slide as long as they wanted. The average amount of total time spent on the lesson slides was a little over 6 min (*M* = 375.55 s, *SD* = 360.42).

##### Learning activity

Between each slide, without the material present, participants were asked to do one of four activities, depending on the condition the participant was randomly assigned to. After the participants were done reading and watching the animated slide, they moved to the next page. Then, participants were shown a slide that prompted them to do the activity relevant to the condition they were in (summarizing, draw, summarize and draw, or no activity). The learning activity was self-paced, meaning the participants could take as much time as needed to complete the activity. The decision to have participants complete the learning activities without the material present was to help reduce differences in the amount of time participants were exposed to the learning material across conditions.

In the control condition, participants were told “Please move to the next slide” after each of the animated slides. The mean amount of total time (i.e., across all four slides) spent on this activity by the participants in the control condition was just over a minute (*M* = 70.60 s, *SD* = 347.48).

In the summary only condition, participants were told “Please provide a summary of the information from the previous animation” after each of the animated slides. A text box was provided for the participants to type in their summary. The mean amount of total time spent on this activity by the participants in the summary only condition was a little over four and a half minutes (*M* = 280.94 s, *SD* = 146.48).

In the draw only condition, participants were told “Please draw a diagram that explains the information from the previous animation. Please upload an image of it below” after each of the animated slides. The mean amount of total time spent on this activity by the participants in the draw only condition was over 11 min (*M* = 681.17 s, *SD* = 453.53).

Lastly, in the draw and summary condition, participants were first told “Please draw a diagram that explains the information from the previous animation. Please upload an image of it below” then “Please provide a summary of the information from the previous animation” after each of the animated slides. The mean amount of total time spent on this activity by the participants in the summary and draw condition was a little over 13 min (*M* = 788.98, *SD* = 299.18).

Participants were not given any prior training on how to complete either the drawing or the summarizing activities. This was done because the purpose of this study was to understand how integrating these activities into an unguided learning environment, seen in many asynchronous learning environments, could impact learning.

The drawings and summaries were scored for whether key ideas presented in the lesson were also presented in the output students created, which we used as a way to measure the quality of the activity. The rubric for scoring the drawings and summaries is included in Appendix A and mean scores for each group are presented in Table 1. Each activity response could earn from four to nine points based on how many main ideas were present in the output, for a total of 24 points across the entire lesson. Two researchers scored each drawing and summary independently; then all disagreements were resolved through discussion until 100% agreement was met. The intraclass correlation coefficient (ICC) demonstrated high reliability in the scoring between the two scorers, ICC = 0.87 (confidence interval: 0.85–0.88). The mean scores for each group are presented in Table 1.

**Table 1:** Mean (and standard deviation) of drawing and summarizing activity scores for Experiment 1.

	Draw only	Summarize only	Summarize and Draw	
			Drawing	Summary
Task 1 Scores: 0–5	3.94 (1.62)	3.13 (1.33)	3.67 (1.62)	3.02 (1.17)
Task 2 Scores: 0–3	1.80 (1.04)	2.54 (0.83)	1.37 (1.08)	2.43 (0.91)
Task 3 Scores: 0–9	3.33 (1.97)	4.87 (2.12)	2.76 (1.70)	4.61 (1.84)
Task 4 Scores: 0–6	1.54 (1.35)	2.91 (1.16)	0.80 (1.08)	2.70 (1.14)

##### Distractor task

The distractor task was used to reduce the amount of rehearsal participants engaged in between the lesson and the posttest. Participants were told to watch a video displaying a compilation of funny dog videos. They were told to pay attention as they would have to answer a question about the video on the next page. The video was 2 min and 21 s and can be found here: https://www.youtube.com/watch?v=kMhw5MFYU0s&ab_channel=It%27sCompilated. Participants were not allowed to move forward in the lesson until the video was done. Once the video was completed, participants were prompted “Please explain your favorite dog fail from the last video you watched.” A text box was provided for participants to type in their answer. Participants were not given any time limit to answer this question, which took on average a little over 4 min (*M* = 246.94 s, *SD* = 893.05).

##### Posttest

The posttest consisted of seven open-ended questions intended to assess the participants’ ability to transfer the information from the lesson to new questions. These questions were: (1) “Based on the lesson you saw, please explain how greenhouse gases work.” (2) “What prevents infrared radiation from leaving the Earth’s atmosphere?” (3) “How would planting more trees/plants affect the temperature of the atmosphere?” (4) “What is a reason that the climate on Earth might show a decrease in temperature?” (5) “Why does your skin feel warm when you step out into the sunlight?” (6) “How would Earth’s atmosphere be different if the atmosphere contained only nitrogen and oxygen?” (7) “How could we decrease the Earth’s temperature without changing the amount of carbon dioxide or methane (greenhouse gases) in the atmosphere?” McDonald’s omega was 0.71, which is considered acceptable reliability. We chose to use omega rather than alpha as the assumptions of alpha tend to be overly stringent for the type of applied research in this study and thus omega is a better measure of reliability (Hayes and Coutts, 2020).

Participants were instructed that they would have 90 s to answer each question, that they would not be able to move forward before the 90 s were done, and they would be auto advanced to the next question after the 90 s were complete. This time limit was used to standardize the testing to determine what participants would most easily be able to access from their prior learning as well as to discourage participants from skipping through the questions too quickly without thought. In pilot testing the material, in which participants had unlimited time to answer each question, 90 s was deemed to be enough time for participants to think about the material and come up with a thoughtful answer, but not too long for participants to lose focus in the task. Our justification for using a timed test in this study is that we wanted to standardize the length and pace of each segment of the experimental session in order to provide better procedural control within the online format of the study, and we wanted to be consistent with dozens of previous value-added studies involving the design of multimedia lessons that successfully used timed tests (Mayer, 2021). The usefulness of this format is demonstrated by its effectiveness in distinguishing between groups as indicated by significant differences in test scores across two experiments.

**Figure 2:**
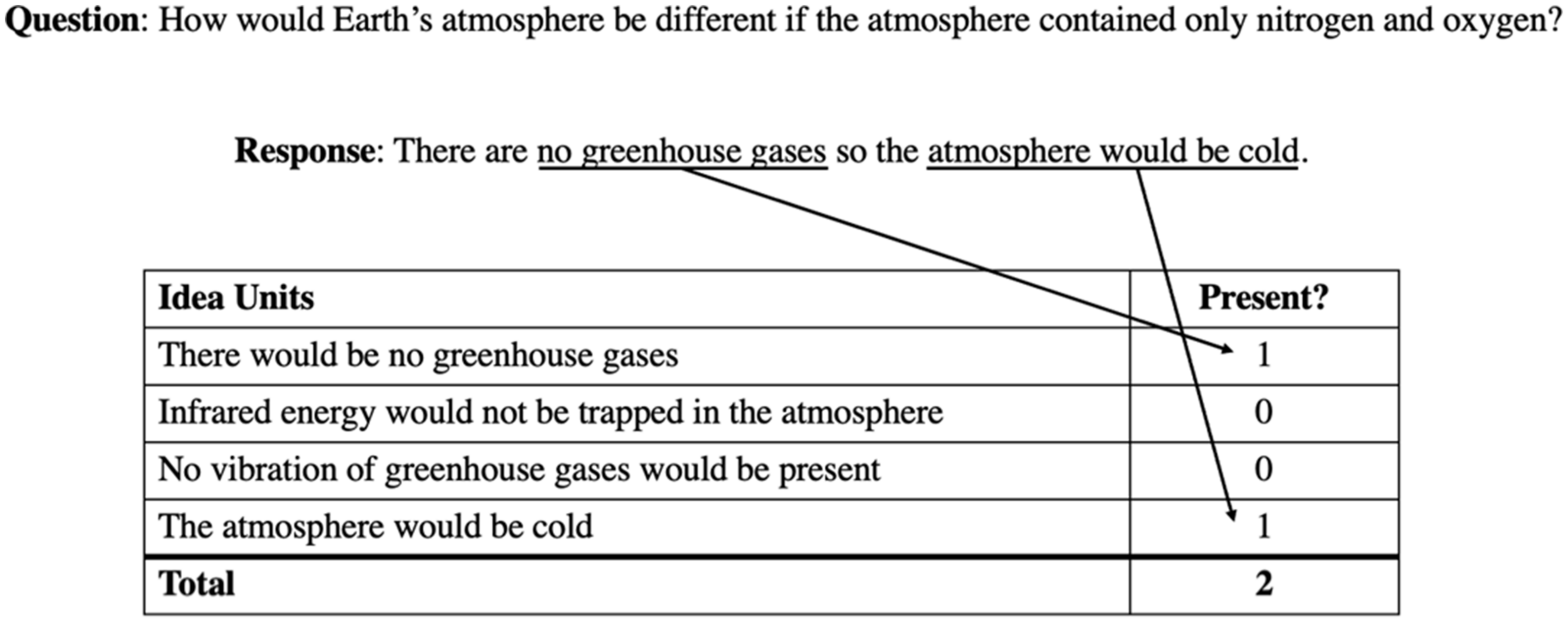
Example of posttest grading.

To score the posttest, a rubric was used to assess how many useful idea units were present in the participants’ responses to the posttest questions (see Appendix B). For each question, there were multiple idea units that could be present in a response; for every idea unit that was present in their response, the participant received a point. This means that participants did not need to list all idea units to have a correct answer nor were they expected to generate all the possible acceptable idea units. For an example of how each question was graded, see Figure 2.

This rubric scheme was used to determine how many useful idea units a learner could generate within a time limit rather than the degree to which their answer was correct. This allowed us to have a better assessment of how deeply participants understood the material presented during the lesson. Scoring the test in this way allows us to assess a range of relevant knowledge, varying from simple factoids drawn directly from the lesson itself to inferences made based on the factoids. With multiple-choice questions or a grading scheme that is more binary, it likely would have been more difficult to distinguish between conditions as deeper knowledge would be harder to assess, which would be less useful in understanding the benefits of these activities for real-life learning. This type of grading scheme has been used in other studies investigating the use of generative learning activities in as well (e.g., Lawson and Mayer, 2021).

With this rubric, participants could earn up to a total of 35 possible points across the seven questions, ranging from four to seven points per question. The rubric for scoring the posttest responses is included in Appendix B, which lists each idea unit for each test item. Two researchers scored each posttest responses independently, then all disagreements were resolved through discussion until 100% agreement was met. The intraclass correlation coefficient (ICC) demonstrated high reliability in the scoring between the two scorers, ICC = 0.94 (confidence interval: 0.93–0.94). Although the total possible number of points is 35 across the seven questions, we did not expect learners to generate all or even most of the acceptable answers as in any divergent thinking task. Thus, it is inappropriate to calculate a percent correct measure, and rather we focus on the total number of acceptable answers generated as our measure of learning outcome. This approach is consistent with dozens of previous studies using open-ended written test items in multimedia design research (Mayer, 2021).

##### Postquestionnaire

The postquestionnaire included seven questions to assess the participants’ experience with the lesson. The first five questions were rated on a 5-point Likert scale from “Strongly Disagree” to “Strongly Agree.” These questions included: (1) “I enjoyed this lesson.” (2) “The topic of this lesson was interesting to me.” (3) “I would like to learn from more lessons like this.” (4) “I felt as though the way this lesson was taught was effective for me.” and (5) “I thought that it was difficult to learn this lesson online.” The sixth question was rated on a 5-point Likert scale from “Very Easy” to “Very Difficult” and asked, “How difficult was the material in this lesson for you?” The last question was rated on a 5-point Likert scale from “Very Little Effort” to “Very High Effort” and was “How much effort did you exert during this lesson?”

#### Procedure

Participants signed up for the study via an online system and were sent an email with the Qualtrics link to the study the morning of the experiment. They were instructed to finish the study by the end of the day and to do the entire study at one time. First, participants read through the consent and agreed to participate. Once they did, they read the instructions and practiced submitting a photo to the Qualtrics page. Once done with that, participants completed the prequestionnaire page. Then, they were randomly assigned to one of the four conditions and completed the lesson and the learning activities for their assigned group. After the lesson was complete, participants watched the distractor video and answered the distractor question. After that, they were taken to the page to complete the posttest questions. Each question was displayed for 90 s and then auto advanced. Once done with that, participants completed the postquestionnaire at their own pace, were asked if anything did not work in the study, and then thanked for their participation. The entire experiment took under 1 h to complete. We obtained Institutional Review Board (IRB) approval and adhered to guidelines for ethical treatment of human subjects.

### Results

#### Do the groups differ in basic characteristics?

The first task was to make sure the four groups were equivalent in basic characteristics. Means and standard deviations on prior knowledge rating and age for each group are reported in two lines of Table 2, respectively. Concerning perception of subjective background knowledge, there was no significant difference based on learning activity, *F*(3, 199) = 1.90, *p* = 0.132. Concerning age, there was no significant difference based on learning activity, *F*(1, 199) = 0.83, *p* = 0.480. Concerning gender, there was no significant difference based on learning activity, *χ*^2^(3, *N* = 202) = 1.19, *p* = 0.755. From this, we can conclude that the groups were equivalent based on these characteristics.

**Table 2:** Means and standard deviations on key measures for four groups in Experiment 1.

	Group							
Measure	Summary and draw		Draw only		Summary only		Control	
	*M*	*SD*	*M*	*SD*	*M*	*SD*	*M*	*SD*
Prior knowledge Scores: 1–5	2.91	1.03	3.06	1.04	3.29	1.07	3.33	0.91
Age	19.26	1.81	18.83	0.91	19.13	1.54	19.19	1.30
Posttest Scores: 0–35	10.22	4.03	10.15	5.14	11.25*	4.80	9.00	4.53
Enjoyment Scores: 1–5	3.64	0.98	3.48	1.19	3.71	0.99	3.48	1.01
Interest Scores: 1–5	4.17	0.80	3.81	1.25	4.04	0.86	3.78	1.01
More lessons Scores: 1–5	3.57	1.15	3.37	1.30	3.62	1.06	3.30	1.17
Effective Scores: 1–5	3.59	1.19	3.31	1.31	3.73	1.04	3.30	1.13
Online difficulty Scores: 1–5	2.39	0.95	2.71	1.27	2.36	1.28	2.87	1.26
Material difficulty Scores: 1–5	1.96	0.76	2.33	1.12	2.11	1.07	2.26	0.85
Effort Scores: 1–5	2.67	0.97	2.90	0.91	2.65	1.02	2.63	1.09

Asterisk (*) indicates significant difference from control group at *p* < 0.05.

#### Hypothesis 1: Do generative learning activities help students perform better on a posttest?

To test hypothesis 1, we ran a one-way ANOVA with an *a priori* Dunnett *post hoc* test comparing each group to the control on posttest score. Means and standard deviations on posttest score for each group are reported in the third line of Table 2. There was not a significant effect of group, *F*(3, 199) = 2.14, *p* = 0.097. The *a priori* Dunnett test helped reveal if there were any differences between the control group and each of the groups with a generative learning activity, as we predicted. There was a significant difference between the summary only group and the control group, *p* = 0.033, *d* = 0.48, with the summary only condition (*M* = 11.25, *SD* = 4.80) outperforming the control condition (*M* = 9.00, *SD* = 4.53). Additionally, there was not a significant difference between the draw only group (*M* = 10.15, *SD* = 5.14) and the control group, *p* = 0.465, *d* = 0.23. Lastly, there was not a significant difference between the draw and summary condition (*M* = 10.22, *SD* = 4.03) and the control group, *p* = 0.425, *d* = 0.28. These results demonstrate that for an immediate test, summarizing was better than watching the lesson with no activity, but drawing alone and mixing drawing and summarizing did not seem to help more than only watching the lesson. These findings are more consistent with the refined interpretation of generative learning theory, which considers the appropriateness of the activity rather than simply the amount of activity.

#### Hypothesis 2: Does combining two generative learning activities help students learn more than doing one activity?

To test hypothesis 2, we ran another Dunnett test to compare each group to the draw and summary condition. There was no significant difference between the draw and summary group and the summary only group, *p* = 0.536, *d* = 0.23. Additionally, there was no significant difference between the draw and summary group and drawing only group, *p* = 1.00, *d* = 0.02. These results show that combining two generative learning activities does not have an additive effect on immediate posttest performance compared to only completing one generative learning strategy.

#### Do different generative learning activities change the experience of learning the lesson?

As an exploratory analysis, we compared the groups on their ratings of each of the seven learning experience items, using one-way ANOVAs. Means and standard deviations on these items are reported in bottom of Table 2. There was not a significant difference among the groups for ratings of enjoyment, *F*(3, 198) = 0.65, *p* = 0.584; ratings of interest in the material, *F*(3, 199) 1.74, *p* = 0.160; ratings of wanting more similar lessons, *F*(3, 199) = 0.90, *p* = 0.442; ratings of the effectiveness of the lesson, *F*(3, 199) = 1.74, *p* = 0.161; ratings of difficulty learning online, *F*(3, 199) = 1.44, *p* = 0.231; ratings of difficulty with learning the material, *F*(3, 199) = 1.44, *p* 0.231; and ratings of the effort put into learning, *F*(3, 199) = 0.743, *p* = 0.528. Based on these findings, we conclude that the experience of learning was similar regardless of the activity the participants were asked to complete during pauses in the multimedia lesson.

#### Does the quality of performance on the learning activity predict posttest scores?

Another exploratory question from this research is whether the performance on the summaries and drawings predicts performance on the posttest. These findings, although not appropriate for drawing causal conclusions about whether the activity caused learning, may help tell if instructors can use certain learning activities as a way to indicate who may need more help or who will most likely perform well on a test of knowledge. The analyses were done per group, so the data was split based on condition.

First, for the summary only group, a simple regression was run with the predictor being the score of the quality of the summary and the outcome being the posttest. The regression found that summary quality explained a significant proportion of the posttest variance, *R^2^* = 0.49, *F*(1, 53) = 16.28, *p* < 0.001. This indicated that for the summary only group, as participants included more key elements in their summaries, the better they performed on the posttest. Relevant statistics are reported in Table 3.

**Table 3:** Regression statistics for Experiment 1.

Condition	*B*	Beta	*t*	*p*
Draw only	0.420	0.594	5.01	<0.001
Summary only	0.443	0.485	4.03	<0.001
Summarize and draw				
Draw Score	0.227	0.295	2.13	0.039
Summarize Score	0.253	0.357	2.583	0.013

Next, for the draw only group, a simple regression was run with the predictor being the score of the quality of the drawing and the outcome being the posttest. The regression found that drawing quality explained a significant proportion of the posttest variance, *R^2^* = 0.35, *F*(1, 46) = 25.08, *p* < 0.001. This indicates that for the draw only group, as participants included more key elements in their drawings, the better they performed on the posttest. Relevant statistics are reported in Table 3.

Lastly, for the draw and summary group, a simple regression was run with the predictors being the score of the quality of the summary and score of the quality of the drawing and the outcome being the posttest. The regression found that the quality of the responses on the learning tasks explained a significant portion of the posttest variance, *R^2^* = 0.44, *F*(1, 45) = 5.01, *p* = 0.011. Both the quality of the drawing (*p* = 0.039) and the quality of the summary (*p* = 0.013) were strong predictors of posttest scores. This means that the more elements included in both the drawings and the summaries, the better participants did on the posttest. Relevant statistics are reported in Table 3.

### Discussion

The main empirical contribution of Experiment 1 is that summarizing only helped students learn better than simply viewing the learning material but drawing only or drawing and summarizing together did not help learning for this particular lesson. Additionally, the experience of learning from a control lesson and the experience of learning with generative learning activities is perceived similarly by learners, so there is little cost to adding generative learning activities to a lesson. Lastly, this research discovered that the quality of the summaries and drawings was prognostic in identifying who would likely do better on an immediate posttest.

## Experiment 2

Experiment 1 showed some benefit for learning when students summarized during pauses in a multimedia lesson involving animation with short text captions; however, immediate tests may not demonstrate the benefits of generative learning in the most effective way (Brown et al., 2014; Dunlosky et al., 2013b; Lawson and Mayer, 2021). The benefits of generative learning activities may be better detected on a delayed test. Thus, the purpose of Experiment 2 was to replicate Experiment 1 with a delayed test, to understand if delayed testing shows similar effects of adding generative learning activities to a multimedia lesson involving annotated animation.

### Method

#### Participants and design

The participants were 130 undergraduate students recruited from a university in southern California through the university’s psychology subject pool. The mean age of these participants was 19.45 years (*SD* = 1.60). Of the 130 participants, 96 identified as women, 31 identified as men, and three did not report their gender identity. As in Experiment 1, the experiment used a one-way between-subjects design with four levels, including a summary only condition with 34 participants, a draw only condition with 26 participants, a draw and summary condition with 36 participants, and a control condition with 34 participants. Sample size was based on a power analysis as in Experiment 1 and was determined to be slightly underpowered. According to a *post hoc* analysis, the power achieved in this test was 0.61. Results should be interpreted with this power issue under consideration.

#### Materials

All of the materials were provided online through Qualtrics, included instructions, and the same as Experiment 1.

##### Instructions

The instructions were essentially the same as Experiment 1. The only difference was that all participants were told that they would complete the study in 2 separate sessions, with a week delay between part 1 and part 2.

##### Prequestionnaire

The prequestionnaire was the same as Experiment 1.

##### Multimedia lesson

The multimedia lessons were the same from Experiment 1. Participants were allowed to stay on each slide as long as they wanted. The mean amount of total time spent on the lesson slides was a little under 7 min (*M* = 418.44 s, *SD* = 716.59).

##### Learning activity

The generative learning activities were the same as Experiment 1. The mean amount of total time spent on this activity by the participants in the control condition was a few seconds (*M* = 8.71 s, *SD* = 6.96). The mean amount of total time spent on this activity by the participants in the summary only condition was a little over 5 min (*M* = 323.34 s, *SD* = 252.86). The mean amount of total time spent on this activity by the participants in the draw only condition was almost 14 min (*M* = 824. 29 s, *SD* = 991.15). The mean amount of total time spent on this activity by the participants in the draw and summary condition was a little over 16 min (*M* = 983.34 s, *SD* = 520.82).

Two researchers scored each summary and each drawing independently; then all disagreements were resolved through discussion until 100% agreement was met. The intraclass correlation coefficient (ICC) demonstrated high reliability in the scoring between the two scorers, ICC = 0.84 (confidence interval: 0.81–0.86). The mean scores for each group are presented in Table 4.

**Table 4:** Mean (and standard deviation) of drawing and summarizing activity scores for Experiment 2.

	Draw only	Summarize only	Summarize and Draw	
			Drawing	Summary
Task 1 Scores: 0–5	3.07 (1.38)	2.57 (1.60)	2.75 (1.42)	2.89 (1.16)
Task 2 Scores: 0–3	1.41 (1.01)	2.20 (0.85)	1.64 (1.05)	2.62 (0.55)
Task 3 Scores: 0–9	3.04 (2.08)	4.74 (1.97)	3.25 (2.07)	4.67 (1.97)
Task 4 Scores: 0–6	1.43 (1.29)	2.89 (1.44)	1.26 (1.46)	3.15 (1.23)

##### Posttest

The posttest was the same as Experiment 1. McDonald’s omega for reliability was 0.70, which once again is considered acceptable. Two researchers scored each posttest answer independently; then all disagreements were resolved through discussion until 100% agreement was met. The intraclass correlation coefficient (ICC) demonstrated high reliability in the scoring between the two scorers, ICC = 0.87 (confidence interval: 0.84–0.89).

##### Postquestionnaire

The postquestionnaire was the same as Experiment 1.

#### Procedure

Participants signed up for the study via an online system and were sent an email with the Qualtrics link to the study the morning of the day they had signed up to participate in session 1. They were instructed to finish this portion of the study by the end of the day and to do the entire session at one time. First, participants read through the consent and agreed to participate. Once they did, they read the instructions and practiced submitting a photo to the Qualtrics page. Once done with that, participants completed the prequestionnaire page. Then, they were randomly assigned to one of the four conditions to complete the lesson and the learning activities. Once done with that, they were thanked for participating in part 1 and reminded to complete part 2 in a week’s time.

A week later, the same participants were sent the Qualtrics link to the second session of the study. They were sent the link in the morning and told to complete this session by the end of the day they had signed up and to complete the session all at one time. When they clicked on the link, they were taken to the page to complete the posttest questions. Each question was displayed for 90 s and then auto advanced. Once done with that, participants completed the postquestionnaire once again, to see if there were any large differences between responses. They were also asked if anything did not work in the study then thanked for their participation. The entire experiment took under 1 h to complete, across both sessions. We obtained Institutional Review Board (IRB) approval and adhered to guidelines for ethical treatment of human subjects.

### Results

#### Do the groups differ in basic characteristics?

The first task was to make sure the four groups were equivalent in basic characteristics. Means and standard deviations of prior knowledge rating and age are reported in the top two lines of Table 5. Concerning subjective perception of background knowledge, there was no significant difference in mean rating among the groups, *F*(3, 126) = 0.23, *p* = 0.878. Concerning age, there was no significant difference of reported age among the groups, *F*(1, 126) = 1.23, *p* = 0.302. Concerning gender, there was no significant difference in the proportion of men and women across the groups, *χ*^2^(3, *N* = 130) = 7.96, *p* = 0.241. From this, we can conclude that the groups were equivalent on these basic characteristics.

**Table 5:** Means and standard deviations on key measures for four groups in Experiment 2.

	Group							
Measure	Summary and draw		Draw only		Summary only		Control	
	*M*	*SD*	*M*	*SD*	*M*	*SD*	*M*	*SD*
Prior knowledge Scores: 1–5	3.00	0.96	3.19	1.02	3.09	1.06	3.03	0.83
Age	19.33	1.10	19.77	2.05	19.68	1.80	19.09	1.40
Posttest Scores: 0–35	7.14	3.78	5.35	3.11	7.35*	5.19	5.00	3.33
Enjoyment Scores: 1–5	3.63	0.77	3.58	0.76	3.53	0.90	3.24	1.00
Interest Scores: 1–5	3.94	0.80	3.58	1.10	3.85	0.93	3.65	0.85
More lessons Scores: 1–5	3.46	1.04	3.15	1.05	3.76	0.87	3.50	0.93
Effective Scores: 1–5	3.29	1.02	3.12	1.07	3.21	1.01	2.91	1.00
Online difficulty Scores: 1–5	2.40	1.22	3.15	1.16	2.74	1.21	2.74	1.19
Material difficulty Scores: 1–5	2.49	0.98	2.62	0.90	2.59	0.82	2.59	0.82
Effort Scores: 1–5	2.80	0.83	2.958	0.81	2.82	0.97	2.44	0.96

Asterisk (*) indicates significant difference from control group at *p* < 0.05.

#### Hypothesis 1: Do generative learning activities help students perform better on a posttest?

To test hypothesis 1, we ran a one-way ANOVA on posttest scores with an *a priori* Dunnett *post hoc* test comparing each generative activity group to the control group. Means and standard deviations on the posttest for each group are reported in the third line of Table 5. There was a significant effect of group in the one-way ANOVA, *F*(3, 126) = 2.14, *p* = 0.032. An *a priori* Dunnett test helped reveal whether there were any differences between each of the generative learning activity groups and the control group. There was a significant difference between the summary only group (*M* = 7.35, *SD* = 5.19) and the control group (*M* = 5.00, *SD* = 3.33) favoring the summary only group, *p* = 0.044, *d* = 0.54. However, there was not a significant difference between the draw only group (*M* = 5.35, *SD* = 3.11) and the control group, *p* = 0.976, *d* = 0.11. Lastly, there was not a significant difference between the draw and summary group (*M* = 7.14, *SD* = 3.78) and the control group, *p* = 0.069, *d* = 0.60. These results replicate Experiment 1 by demonstrating that for a delayed test, summarizing led to better learning outcomes than a control, but drawing did not, and combining summarizing and drawing did not improve learning for this particular lesson. However, due to the underpowered nature of this experiment and the effect size of the combined draw and summary group compared to the control group, if there was enough power, there may have been a significant effect demonstrating the effectiveness of combining the conditions compared to the control condition.

#### Hypothesis 2: Does combining two generative learning activities help students learn more than doing one activity?

Another Dunnett test was used to compare draw and summary group with each of the other generative learning groups. There was no significant difference between the draw and summary group and the summary only group, *p* = 0.992, *d* = 0.05. Additionally, there was no significant difference between the draw and summary group and the draw only group, *p* = 0.202, *d* = 0.52. In a replication of Experiment 1, these results suggest that combining two generative learning activities does not have simple additive effects on a delayed posttest compared to only completing one generative learning activity. However, once again due to the underpowered nature of this experiment and effect size of the combined draw and summary group compared to the control group, if there had been enough power, there might have been a significant effect demonstrating the effectiveness of combining the conditions in comparison to the draw condition but not the summarize condition only.

#### Do different generative learning activities change the experience of learning the lesson?

As an exploratory analysis, we compared the groups on mean ratings of the seven learner experience items, using one-way ANOVAs. Means and standard deviations on each item by the four groups are reported in Table 5. There was not a significant difference among the groups on ratings of enjoyment, *F*(3, 124) = 1.31, *p* = 0.275; ratings of interest in the material, *F*(3, 125) = 1.10, *p* = 0.350; ratings of wanting more similar lessons, *F*(3, 124) = 1.89, *p* = 0.134; ratings of the effectiveness of the lesson, *F*(3, 125) = 0.86, *p* = 0.467; ratings of difficulty learning online, *F*(3, 125) = 1.98, *p* = 0.121; ratings of the difficulty of the material, *F*(3, 125) = 0.14, *p* = 0.937; or ratings of the effort put into learning, *F*(3, 125) = 1.40, *p* = 0.247. Based on these findings and consistent with Experiment 1, we conclude that the learning experience of learning was similar among the four groups.

#### Does the quality of performance on the learning activity predict posttest scores?

Another exploratory question from this research is whether the performance on the summaries and drawings predicted performance on the delayed posttest. The analyses were done per group, so the data was split based on condition.

First, for the summary only group, a simple regression was run with the predictor being the score of the quality of the summary and the outcome being the posttest score. The regression found that summary performance explained a significant proportion of the posttest variance, *R^2^* = 0.33, *F*(1, 32) = 15.52, *p* < 0.001. This indicated that for the summary only group, as participants included more key elements in their summaries, the better they performed on the posttest, yielding a prognostic pattern, which is consistent with Experiment 1. Relevant statistics are reported in Table 6.

**Table 6:** Regression statistics for Experiment 2.

Condition	*B*	Beta	*t*	*p*
Draw only	0.102	0.070	0.41	0.509
Summary only	0.559	0.571	3.94	<0.001
Summarize and draw				
Draw Score	0.014	0.017	0.10	0.922
Summarize Score	0.199	0.201	1.14	0.263

Next, for the draw only group, a simple regression was run with the predictor being the score of the quality of the drawing and the outcome being the posttest score. The regression found that drawing performance did not explain a significant proportion of the posttest variance, *R^2^* = 0.02, *F*(1, 24) = 0.45, *p* = 0.509. This indicates that for the draw only group, the number of elements included in the drawing did not predict posttest performance, which is inconsistent with Experiment 1. Relevant statistics are reported in Table 6.

Lastly, for the draw and summary group, a simple regression was run with the predictors being the score of the quality of the summary and score of the quality of the drawing and the outcome being the posttest score. The regression found that the quality of the responses on the learning tasks did not explain a significant portion of the posttest variance, *R^2^* = 0.04, *F*(1, 33) = 0.73, *p* = 0.488. Both the quality of the drawing (*p* = 0.922) and the quality of the summary (*p* = 0.263) were not predictors of posttest scores. This means that the number of elements included in the drawing and summary did not predict posttest performance, which is inconsistent with Experiment 1. Relevant statistics are reported in Table 6.

### Discussion

As in Experiment 1, the main empirical contribution of Experiment 2 is that adding prompts to engage in summarizing during pauses in this particular multimedia lesson aided learning, whereas prompts to engage on drawing or to both draw and summarize did not. However, the effect size found comparing the combined condition to no activity may suggest that there may have been a benefit of combining activities, if the study was not underpowered Additionally, combining two generative learning activities in this lesson was not more effective than simply having summarizing as a single activity. Once again however, the effect size found comparing the combined group to the draw group may suggest that there may have been a benefit, if the study was not underpowered. Lastly, the quality of summaries was predictive of posttest score, but the quality of drawing was not. More work needs to be done to understand how activities, particularly summarizing, may be a prognostic measure of how students might do on a test.

## General discussion

### Empirical contributions

The primary new empirical contribution of this work supports the more refined interpretation of generative learning theory that is focused on in which specific situations untrained generative learning activities would best promote generative processing. This research found that asking students to generate verbal summaries during pauses in an online, self-paced multimedia lesson led to better transfer scores on immediate and delayed posttests compared to a control. This suggests that when individuals learned using an animated online multimedia lesson, summarizing was useful in encouraging students to engage in generative processing and translate the representation meaningfully. However, asking students to generate drawings during pauses in an online, self-paced multimedia lesson did not lead to better transfer in an immediate or delayed posttest compared to a control. This suggests that drawing may not have been useful in encouraging students to engage in generative processing and translating the representation meaningfully when learning from the online multimedia lesson without any prior training in generative drawing.

In addition, combining drawing and summarizing was not more beneficial to learning than summarizing alone, drawing alone, or a control. However, in Experiment 2, the effect size of the difference between the combined drawing and summarizing condition compared to the control condition may suggest that, if the study was not underpowered, the study would have found a benefit of combining activities, at least in comparison to not engaging in any activities. Additionally, there may have been a positive effect of combining activities over only drawing, had this study not be underpowered. Despite this potential problem, even with enough power, it does not seem as though combining drawing and summarizing created an additive effect of generative learning that would have had stronger effects than only drawing and only summarizing.

This research adds to the literature on generative learning activities in several ways. First, it demonstrates that even without any prior training on the technique and in an environment that may be less motivating to engage in translation of mental representations, summarizing is an effective strategy for encouraging generative learning. This finding is beneficial to add to the literature because it demonstrates that even without guidance from an instructor, summarizing can be used to encourage generative processing and benefit deeper understanding of the material.

Second, drawing did not seem to be effective at encouraging generative learning from this lesson, with one potential conclusion being because the multimedia presentation was highly visual and thus less motivating for students to translate the visual representation of the material into their own representation. Because the lesson was highly visual to begin with, drawing may not have encouraged as deep of generative processing because learners could simply copy aspects of the presented animation without having to build connections between visual and verbal representations. As suggested in prior literature, for generative learning activities to be effective, learners must engage with the material in a meaningful way that causes them to select relevant visual and verbal information, build visual and verbal representations, and mentally integrate them (Lawson and Mayer, 2021). If learners did not need to spend time thinking about how to translate their knowledge into a drawing that was unique from the presented visualizations, they likely would not need to engage in generative processing and therefore would not benefit from this activity. This research helps establish that one of the boundary conditions in using drawing may be the type of lesson students are learning from, specifically how visual the information is.

Alternatively, it may have been the case that drawing was not effective in encouraging generative learning from this lesson because participants were not trained in how to create drawings that would enhance generative learning. As previously investigated, both summarizing and drawing may be more effective when students are given training in how to implement them (e.g., Fiorella and Mayer, 2015, 2016; Van Meter and Garner, 2005). Many students have experience and training with summarizing, as this is a common technique used in education. However, drawing is less commonly used, and thus may have been more useful if training was provided before or during the course of this study. As such, future research should investigate whether training in drawing can increase the benefits of using drawing as a generative learning activity for online multimedia lessons.

This series of studies also demonstrated that the quality of a summary completed while learning may help predict whether learners will do well on a test of the material. In both experiments, providing a thorough summary was positively related to learners understanding of the material. However, this was true only when they were not asked to do another task on top of summarizing. Additionally, drawing quality was not consistently prognostic of how well learners would perform on the posttest; when tested with an immediate test, there was a relationship between drawing quality and posttest performance but this relationship went away when a delayed posttest was used. This is in contrast to prior work demonstrating that drawing can be a prognostic activity (Mason et al., 2013; Schmeck et al., 2014; Schwamborn et al., 2010). In these studies, drawings were constructed based on text-only lessons, so it is possible that boundary condition for drawing to be a prognostic activity is that the lesson involves text rather than text and graphics. Additionally, drawing may not have been predictive of learning because of the lack of training on how to create drawings that encouraged generative thinking.

### Theoretical contributions

The major theoretical contribution of this study is to support a refined version of generative learning theory that considers the appropriateness of learning activity for a particular learning environment rather than assuming that just prompting generative learning through any activity is beneficial. Generative learning theory suggests that if learners engage in appropriate cognitive processing during learning, they should have a better understanding of the material than someone who does not engage in these types of processes. This research contributes to generative learning theory by showing that encouraging this type of processing requires a nuanced understanding of how learners may experience a particular lesson.

A potential reason for the pattern of results in which summarizing benefitted learners while drawing did not, may be due to differences in how these activities were encouraging generative processing when used with animated lessons. Different generative learning activities use different tactics for priming appropriate processes during learning, such as verbal tactics when engaging in summarizing and visual tactics when engaging in drawing. It may be the case that the visual representation in multimedia lessons, like the one in this set of experiments, provide rich visual representations so creating a new drawing was not required and thus did not benefit learning. This research demonstrates the potential of generative learning activities to affect how students learn from online, self-paced multimedia lessons.

### Practical contributions

This series of studies presents two important practical contributions. The first contribution focuses on the fact that it is vital to think about how students are going to experience and work through a lesson when deciding what types of activities to add to a lesson, especially when an instructor is not present to train learners in conducting learning activities. This study demonstrated that adding more prompts to engage students in untrained generative learning does not necessarily benefit learners and only certain types of activities, specifically an activity that was more likely to promote translation of information, had benefits for learning. When designing lessons with generative learning activities, instructors must first put thought into how a student may experience the lesson and what kinds of cognitive benefits the students would get from engaging in the activity in the specific lesson before incorporating the activity into the lesson. For example, having a visual generative learning strategy may be more beneficial in promoting generative processing in situations where learning is occurring from text-based environments, but not promote generative processing as well in an animation-based lesson or video-based lesson, particularly when an instructor is not there to monitor a student’s progress. Additionally, it is beneficial to think about whether students may need prior training in a learning activity in order to benefit from engaging in the activity.

Second, this study demonstrates that adding generative activities to multimedia lessons does not change how students experience the lesson, even when the lesson is self-guided and self-paced. As suggested by this research, there is very little cost to adding generative activities to lessons, and in some cases, the payoff is high. Including activities that are likely to increase generative processing can increase students’ longer-term understanding of the material without making the lesson feel more difficult, less engaging, or less enjoyable.

### Limitations and future directions

One limitation of this study is the fact that the lesson was very short and isolated. In real experiences, most learning occurs over weeks or years and includes material that builds upon itself. This lesson was a one-time lesson that took less than 15 min to complete. This discrepancy makes the findings difficult to generalize to more realistic learning settings. Future research should aim to understand how generative learning activities can operate in more naturalistic classroom settings with material that builds off prior material learned.

Furthermore, this study presented material that was generally simple and straightforward. This may have reduced the impact that generative learning activities could play in helping students understand the material. With more difficult material, generative learning activities and combining activities may play a larger role in helping students to understand the material more deeply. Future research should investigate how using generative learning activities and combining activities can impact learning from difficult lessons.

Additionally, as discussed previously, these studies were underpowered, likely due to the impact of recruiting participants during COVID-19 restrictions. As such, it is more likely that Type II error has occurred, although we have attempted to point out when this may be the case throughout the paper. Future research should aim to replicate these studies with larger sample sizes to understand whether the results replicate.

A potential reason for the results finding that summarizing was effective but not drawing may come from the nature of the posttest. The posttest itself asked students to verbally explain the material through written responses rather than through pictorial responses. The mismatch in verbal response required on the posttest and the pictorial response required in the drawing condition may have lowered the effectiveness of drawing as a generative learning strategy in comparison to the match for the verbal response of the posttest and summarizing condition. Additionally, the posttest was timed, which may have created a scenario in which the responses to the questions were less complete than if the test was untimed. More work should be done investigating how participants perform on more visually-based posttests after drawing to determine if the mismatch in tasks is a reason for the poorer performance of the drawing condition in this study as well on tests that do not have time limits.

Another potential limitation is that within the combined condition, learners were always asked to first draw then summarize, and this was not counterbalanced nor were the conditions integrated. This limits the generalizability of this study as it only tested the combination of activities in one direction. Additionally, the research prompts did not integrate drawing and summarizing into one activity, but rather had participants engage in the activities sequentially. As such, future research should look at how changing the order of how students engage in generative learning activities or integrating the two generative learning activities into one activity could impact their learning.

We did not manipulate the presence or absence of animation in this study, so we cannot definitively attribute the ineffectiveness of learning by drawing to the presence of animation in the lesson. Future research should address this issue. Another direction for future research is to determine the degree to which students in the summarizing condition extract the captions verbatim and use them for their answers to test questions. Furthermore, given the differences in time that students spent on the generative learning activities in the two experiments, future work should focus on analyzing how students carry out their activities perhaps through thinking aloud protocols.

Future research should continue to explore how different types of generative learning activities can be effectively combined to produce additive effects. From Ponce et al.’s (2018) study, it is evident that combining generative activities in a lesson can have an additive effect, but this is not true for all cases. It is important to consider how well the two activities can be coordinated with one another and the lesson itself. Additionally, cognitive load could be a factor that influences the impact of combining generative learning activities on learning and should be investigated further. In a similar vein, future research should investigate how combining generative learning activities may be more successful if there is a better fit between the activities used and the material presented. This would allow us to understand the results more clearly from this experiment. Lastly, to identify for whom specific generative learning activities work best, it would be particularly beneficial to research the interaction of individual differences and generative learning activities.

## Conclusion

This study investigated both the use of generative learning activities in online multimedia lessons and the effectiveness of combining multiple generative learning activities together to enhance learning. In a series of two studies, summarizing was found to be effective in helping students understand the lesson better compared to a control in both an immediate (Experiment 1) and delayed test (Experiment 2), possibly because it was better able to encourage students to engage in appropriate generative processing during learning (e.g., connecting verbal and visual representations). However, drawing alone was not more effective than a control on either the immediate test or the delayed test for this lesson, potentially because it was less able to encourage students to engage in appropriate generative processing (e.g., connecting verbal and visual representations). Lastly, combining activities was not more effective than a control on either an immediate or delayed test. This study showed that summarizing is a beneficial tool in learning from online, self-paced multimedia lessons, and more research needs to be done to determine the situations in which drawing or combining drawing and summarizing can enhance learning.

## Data Availability

The raw data supporting the conclusions of this article will be made available by the authors, without undue reservation.
