# Supplementary material for: Generative learning activities for online multimedia learning: when summarizing is effective but drawing is not
Source: Front Psychol. 2024 Sep 2;15:1452385. doi: 10.3389/fpsyg.2024.1452385 (PMC11402827; doi:10.3389/fpsyg.2024.1452385)
Supplement: Supplementary file 1 [file Presentation_1.pdf]

Appendix A

Grading Rubric for Summaries and Drawings

Activity 1: Draw or summarize slide 1

Themes:
- Energy comes from the sun
- Energy is reflected off Earth
- Energy is absorbed by Earth
- Light colors reflect energy
- Dark colors absorb energy

Could earn up to 5 points

Activity 2: Draw or summarize slide 2

Themes:
- Energy is absorbed by Earth
- Absorbed energy heats up the Earth’s surface
- Absorbed energy emitted as infrared radiation from Earth

Could earn up to 3 points

Activity 3: Draw or summarize slide 3

Themes:
- The air is made up of different molecules
- Some molecules are greenhouse gases (like carbon dioxide and methane)
- Greenhouse gases absorb/interact with infrared radiation
- Greenhouse gases vibrate from infrared radiation
- Vibrating greenhouse gases heat the air around them
- Greenhouse gases give off infrared radiation
- Infrared radiation can go back down to Earth
- Infrared radiation can go to other greenhouse gases
- The cycle can repeat/traps heat

Could earn up to 9 points

Activity 4: Draw or summarize slide 4

Themes:
- More greenhouse gases lead to warmer temperatures
- More greenhouse gases mean more infrared radiation is absorbed
- More greenhouse gases mean more vibration
- More greenhouse gases mean more infrared radiation is given off

- More greenhouse gases mean air gets warmer
- Similar to how crowded room heats up with more people

Could earn up to 6 points

Appendix B

Rubric for Grading Posttest Responses

Q1: Based on the lesson you saw, please explain how greenhouse gases work.

Main Ideas:
- Infrared radiation is released from Earth's surface into the atmosphere
- Greenhouse gases interact with infrared radiation
- Greenhouse gases vibrate when they interact with infrared radiation
- Vibration of greenhouse gases causes heat
- Greenhouse gases release infrared radiation in different directions
- Infrared radiation can go back to Earth, repeating the process
- Infrared radiation can go to other greenhouse gases, repeating the process

Could earn up to 7 points

Q2: What prevents infrared radiation from leaving the Earth's atmosphere?

Main Ideas:
- Infrared radiation is stopped by greenhouse gases in the atmosphere
- Infrared radiation interacts with greenhouse gases
- Infrared radiation can be sent in different directions after interaction with greenhouse gases
- Infrared radiation can go back down to Earth
- Infrared radiation can go outward and hit other greenhouse gases

Could earn up to 5 points

Q3: How would planting more trees/plants affect the temperature of the atmosphere?

Main Ideas:
- Trees absorb carbon dioxide
- Less greenhouse gases in the atmosphere (with lesson CO_2)
- Less greenhouse gases mean less greenhouse gases to absorb infrared radiation
- Less vibration occurs
- Less infrared radiation reflected back to Earth
- More infrared radiation leaving the atmosphere/ not trapped
- Lower temperature

Could earn up to 7 points

Q4: What is a reason that the climate on Earth might show a decrease in temperature?

Could only get points within 1 of these 2 ideas
Main Ideas A:

- Less greenhouse gases in the atmosphere
- Less greenhouse gases means less vibrations
- Less vibrations mean less heat
- Less greenhouse gases means less trapped infrared radiation

Main Ideas B:
- Less sunlight
- Less sunlight means less infrared radiation released from Earth
- Less infrared radiation means less interactions with greenhouse gases
- Less vibrations means less heat

Other less possible but still correct ideas were able to earn points as well

Could earn up to 4 points

Q5: Why does your skin feel warm when you step out into the sunlight?

Main Ideas:
- All skin has pigment, so it is not fully white/reflective
- Anything with some sort of pigment absorb energy
- Energy is absorbed in the skin
- Absorbed energy in the skin means the skin is vibrating

Could earn up to 4 points

Q6: How would Earth's atmosphere be different if the atmosphere contained only nitrogen and oxygen?

Main Ideas:
- No greenhouse gases
- No greenhouse gases to trap infrared radiation in the atmosphere
- No vibration of greenhouse gases
- Cold atmosphere

Could earn up to 4 points

Q7: How could we decrease the Earth's temperature without changing the amount of carbon dioxide or methane (greenhouse gases) in the atmosphere?

Could only get points within 1 of these 2 ideas
Main Ideas A:
- Less sunlight
- Less infrared radiation related from Earth
- Less infrared radiation means less interaction with greenhouse gases and vibrations
- Less vibrations means less heat

Main Ideas B:

- White/light colored planet
- Less infrared radiation is released from Earth
- Less infrared radiation means less interactions with greenhouse gases and vibrations
- Less vibrations mean less heat

Other less possible but still correct ideas were able to earn points as well
